# Tomoelastography for Longitudinal Monitoring of Viscoelasticity Changes in the Liver and in Renal Allografts after Direct-Acting Antiviral Treatment in 15 Kidney Transplant Recipients with Chronic HCV Infection

**DOI:** 10.3390/jcm10030510

**Published:** 2021-02-01

**Authors:** Stephan R. Marticorena Garcia, Christian E. Althoff, Michael Dürr, Fabian Halleck, Klemens Budde, Ulrike Grittner, Christian Burkhardt, Korinna Jöhrens, Jürgen Braun, Thomas Fischer, Bernd Hamm, Ingolf Sack, Jing Guo

**Affiliations:** 1 Department of Radiology, Charité—Universitätsmedizin Berlin, Corporate Member of Freie Universität Berlin, Humboldt-Universität zu Berlin, and Berlin Institute of Health, Charitéplatz 1, 10117 Berlin, Germany; christian.althoff@charite.de (C.E.A.); christian.burkhardt@charite.de (C.B.); thom.fischer@charite.de (T.F.); bernd.hamm@charite.de (B.H.); ingolf.sack@charite.de (I.S.); Jing.Guo@charite.de (J.G.); 2 Department of Nephrology and Medical Intensive Care, Charité—Universitätsmedizin Berlin, Corporate Member of Freie Universität Berlin, Humboldt-Universität zu Berlin, and Berlin Institute of Health, Charitéplatz 1, 10117 Berlin, Germany; michael.duerr@charite.de (M.D.); fabian.halleck@charite.de (F.H.); klemens.budde@charite.de (K.B.); 3 Institute of Biometry and Clinical Epidemiology, Charité—Universitätsmedizin Berlin, Corporate Member of Freie Universität Berlin, Humboldt-Universität zu Berlin, and Berlin Institute of Health, Charitéplatz 1, 10117 Berlin, Germany; Ulrike.Grittner@charite.de; 4 Berlin Institute of Health (BIH), Anna-Louisa-Karsch 2, 10178 Berlin, Germany; 5 Department of Pathology, Charité—Universitätsmedizin Berlin, Corporate Member of Freie Universität Berlin, Humboldt-Universität zu Berlin, and Berlin Institute of Health, Charitéplatz 1, 10117 Berlin, Germany; korinna.joehrens@uniklinikum-dresden.de; 6 Institute for Medical Informatics, Charité—Universitätsmedizin Berlin, Corporate Member of Freie Universität Berlin, Humboldt-Universität zu Berlin, and Berlin Institute of Health, Charitéplatz 1, 10117 Berlin, Germany; juergen.braun@charite.de

**Keywords:** hepatitis C virus, liver fibrosis, kidney transplantation, direct-acting antiviral agents, magnetic resonance elastography, tomoelastography, stiffness

## Abstract

Besides the liver, hepatitis C virus (HCV) infection also affects kidney allografts. The aim of this study was to longitudinally evaluate viscoelasticity changes in the liver and in kidney allografts in kidney transplant recipients (KTRs) with HCV infection after treatment with direct-acting antiviral agents (DAAs). Fifteen KTRs with HCV infection were treated with DAAs (daclatasvir and sofosbuvir) for 3 months and monitored at baseline, end of treatment (EOT), and 3 (FU1) and 12 (FU2) months after EOT. Shear-wave speed (SWS) and loss angle of the complex shear modulus (φ), reflecting stiffness and fluidity, respectively, were reconstructed from multifrequency magnetic resonance elastography data with tomoelastography post-processing. After virus elimination by DAAs, hepatic stiffness and fluidity decreased, while kidney allograft stiffness and fluidity increased compared with baseline (hepatic stiffness change at FU1: −0.14 m/s, *p* < 0.01, and at FU2: −0.11 m/s, *p* < 0.05; fluidity at FU1: −0.05 rad, *p* = 0.04 and unchanged at FU2: *p* = 0.20; kidney allograft stiffness change at FU1: +0.27 m/s, *p* = 0.01, and at FU2: +0.30 m/s, *p* < 0.01; fluidity at FU1 and FU2: +0.06 rad, *p* = 0.02). These results suggest the restoration of mechanically sensitive structures and functions in both organs. Tomoelastography can be used to monitor the therapeutic results of HCV treatment non-invasively on the basis of hepatic and renal viscoelastic parameters.

## 1. Introduction

Hepatitis C virus (HCV) infection is associated with chronic inflammation and is a predisposing factor for liver fibrosis, leading to an accumulation of extracellular matrix proteins [1]. Besides the liver, HCV infection also affects the kidney [2,3,4,5], causing increased morbidity and mortality of kidney transplant recipients (KTRs) compared with patients who have normal renal function [6,7]. Furthermore, immunosuppression commonly applied to KTRs could also lead to a high risk of HCV reactivation and recurrence, resulting in the progression of hepatic fibrosis and, ultimately, a high incidence of liver cirrhosis and hepatocellular carcinoma [8,9]. Direct-acting antiviral agents (DAAs) are very effective in viral eradication, decreasing the risk of hepatocellular carcinoma [10,11,12,13]. As they are very effective in HCV therapy [14,15,16,17], DAAs are recommended by the current Kidney Disease Improving Global Outcomes (KIDGO) guidelines for treating HCV-infected patients with renal disease [18].

In KTRs with HCV infection who are treated with DAAs, the kidney allograft requires attention, as sofosbuvir, one of the commonly used DAAs, is excreted mainly by the kidneys [19]. Although sufficiently safe in dysfunctional native or transplanted kidneys [13,19,20,21,22,23], the influence of DAAs on renal structure and perfusion, reflected by viscoelastic parameters, is largely unknown. The viscoelasticity of soft organs such as the kidneys is known to be sensitive to early changes in extracellular matrix protein accumulation and blood perfusion. However, viscoelastic parameter changes due to DAA treatment have only been reported for the liver. Earlier studies have shown that multifrequency magnetic resonance elastography (MRE) with novel tomoelastography data processing was highly sensitive to structural changes in dysfunctional native [24,25] and transplanted [26] kidneys. On the basis of that work, we hypothesize that the viscoelastic parameters of the transplanted kidney reflect possible early structural and functional changes after DAA treatment that are not detected by other biomarkers such as serum creatinine and proteinuria [25].

The aim of this study was to longitudinally assess the mechanical response of: (i) the liver and (ii) the transplanted kidney to DAA treatment in HCV-infected KTRs by tomoelastography. We also investigated the association of mechanical changes with biochemical markers that are directly related to the functional status of the liver and kidney allografts.

## 2. Materials and Methods

### 2.1. Study Population

In this prospective, single-center study (EudraCT number: 2014-004551-32) [27], KTRs with chronic HCV infection and clinical indication for DAA treatment were recruited at the transplant center of our hospital between December 2015 and July 2016. MRE experiments were approved by our local institutional review board (EA1/075/17, EA1/019/15) and all subjects gave written informed consent.

**Figure 1:**
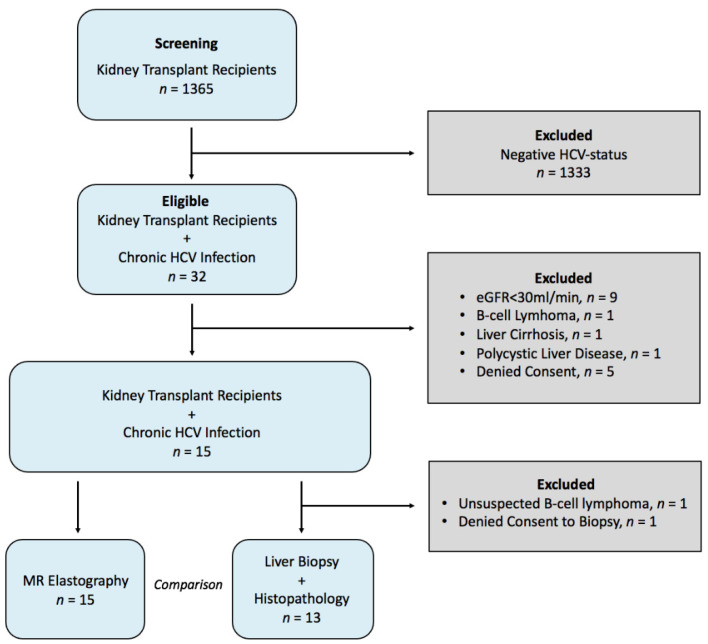
Study flow diagram. eGFR = estimated glomerular filtration rate.

The inclusion criteria were: (i) age at least 18 years; (ii) diagnosed with chronic HCV infection of genotype Ia or Ib, defined by having detectable anti-HCV antibodies and a HCV RNA viral load for more than 3 months; (iii) untreated with, or be non-responding to, other anti-HCV treatment; (iv) having an estimated glomerular filtration rate (eGFR) above 30 mL/min/1.73 m^2^ for more than 12 months (estimated from blood creatinine levels by the Chronic Kidney Disease Epidemiology Collaboration (CKD-EPI) equation [28]). The exclusion criteria were: (i) contraindications to daclatasvir and sofosbuvir, co‑infections such as human immunodeficiency virus or hepatitis B virus, or chronic decompensated liver disease (Child–Pugh class B or C); (ii) polycystic liver or kidney disease; (iii) history of kidney allograft rejection; (iv) history of malignancies; (v) contraindications to magnetic resonance imaging (MRI); and (vi) current participation in other drug trials. A study flow diagram is given in Figure 1.

### 2.2. Study Protocol

**Figure 2:**
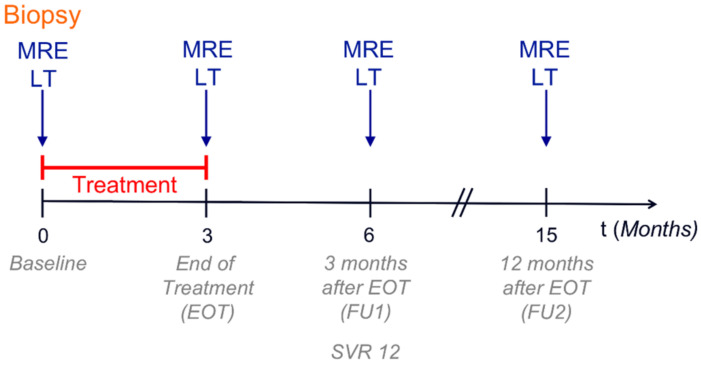
Study design. MRE = multifrequency magnetic resonance elastography; LT = laboratory tests; EOT = end of treatment; SVR = sustained virological response; red bar = treatment period.

Patients received 60 mg daclatasvir and 400 mg sofosbuvir (DAAs) daily over a period of 3 months. HCV RNA and clinical data were assessed according to the study protocol of the DAA safety study of Duerr et al. [27]. Laboratory markers from blood and urine analysis were taken at four time points when MRE was performed: baseline, at the end of treatment (EOT), 3 months after EOT (FU1), and 12 months after EOT (FU2). FU1 also corresponds to a sustained virological response (SVR) after 12 weeks (SVR12). The study timeline is depicted in Figure 2.

### 2.3. Response to Therapy

HCV RNA levels ≤ 15 IU/mL were considered as SVR. Patients with SVR12 were defined as responders, according to [13]. A recurrence of HCV (HCV RNA levels > 15 IU/mL), confirmed by two consecutive positive HCV RNA analyses, was considered as viral relapse. In cases of viral relapse, the treatment with combined daclatasvir and sofosbuvir was extended to 24 weeks.

### 2.4. Tomoelastography

To address the previously reported post-prandial effects on liver viscoelasticity [29,30], all patients were instructed to fast for at least two hours before MRE examinations. To exclude confounding factors such as cholestasis and venous congestion in investigating liver stiffness [31], blood serum bilirubin and the diameter of the inferior vena cava were monitored at all time points using T2-weighted images. Multifrequency MRE was conducted as described in [30]. In brief, three compressed-air-powered actuators were used, two of which were placed posteriorly and a third anteriorly directly above the liver or kidney allograft. The air pressures used in the liver and for the kidney allograft were 0.5 and 0.3 bar, respectively.

All imaging examinations were performed on a 1.5 T MRI scanner (Magnetom Sonata; Siemens, Erlangen, Germany) using a 12-channel phased-array surface coil. Three-dimensional wave fields were acquired using a single-shot, spin-echo planar imaging sequence with flow-compensated motion-encoding gradients (MEG) as detailed in [32]. Eight wave-phase offsets were recorded over a full vibration period. Four vibration frequencies were applied: 30, 40, 50 and 60 Hz in the liver, and 40, 50, 60 and 70 Hz in the kidney allograft. Under free breathing, 11 axial slices with 2.7 × 2.7 × 5 mm^3^ resolution covering the entire liver and 9 paracoronar slices with 2.5 × 2.5 × 2.5 mm^3^ resolution covering the entire kidney allograft along the longitudinal axis were acquired in two and four minutes, respectively. Other MRE parameters for the liver were: repetition time (TR) = 1180 ms; echo time (TE) = 55 ms; parallel imaging with a generalized autocalibrating partial parallel acquisition (GRAPPA) factor of 2; MEG frequency = 47.62 Hz for all mechanical frequencies; and MEG amplitude = 25 mT/m. Other MRE parameters for the kidney allograft were: TR = 1200 ms; TE = 55 ms; parallel imaging with a GRAPPA factor of 2; MEG frequency = 48.45 Hz for vibration frequencies of 40 Hz, 50 Hz and 60 Hz, and 52.41 Hz for a vibration frequency of 70 Hz; MEG amplitude = 25 mT/m.

### 2.5. Data Processing

Tomoelastography parameter reconstruction was performed with the publicly available server-based processing pipeline [33]. We used multifrequency dual elasto-visco inversion (MDEV) for reconstruction of the phase angle of the complex shear modulus (φ in rad) [34] and wavenumber-based MDEV (k‑MDEV) for reconstruction of shear-wave speed (SWS in m/s) [35]. SWS reflects tissue stiffness, while φ is related to the solid–fluid behavior of the tissue. Since larger values of φ (φ > φ/4) are considered to be dominated by fluid tissue properties, φ is also referred to as “fluidity”. Maps of SWS and φ are referred to hereinafter as elastograms. The regions of interest were drawn manually on the basis of the MRE magnitude images and the corresponding elastograms. In kidney allografts, the entire parenchyma, comprising the cortex and medulla, was considered for analysis.

### 2.6. Biopsy and Histological Staging

All percutaneous biopsies were performed under ultrasound guidance by a single interventional radiologist using an Acuson X700 (Siemens, Erlangen, Germany). For each patient, three intercostal biopsies of the right lobe were performed using an 18G Quick-Core^®^ Biopsy Needle (William Cook Europe ApS, Bjaeverskov, Denmark) [36]. One patient with an increased risk of bleeding was treated by transjugular liver biopsy (Liver Access and Biopsy Needle Set, LABS-200-J, 19G; William Cook Europe ApS, Bjaeverskov, Denmark) according to [37]. Biopsy samples with a minimum length of 2.0 cm were obtained and directly conserved in 4% formalin.

Histopathological analyses were performed by a single pathologist with high expertise (more than 20 years) in liver pathology. The modified Scheuer classification [38,39] was used for staging fibrosis and inflammation.

### 2.7. Laboratory Tests

All laboratory values such as viral load (HCV RNA, IU/mL; assayed by quantitative reverse transcription polymerase chain reaction), alanine aminotransferase (ALT), aspartic acid aminotransferase (AST), blood platelet count, total bilirubin, creatinine, and proteinuria were collected at baseline, at EOT, at FU1, and at FU2 (Figure 2). Serological fibrosis scores such as aspartate aminotransferase to platelet ratio index (APRI) [40] and fibrosis-4 (FIB-4) [41], based on blood serum values, were calculated according to the following formulae:

(1) $$\mathrm{APRI}=\frac{\mathrm{AST}\;\left(U/L\right)}{\mathrm{Platelet}\;\mathrm{count}\;\left(10^{9}/L\right)}\;\times \;100$$

(2) $$\mathrm{FIB}–4=\;\frac{\mathrm{Age}\;\left(\mathrm{years}\right)\times \;\mathrm{AST}\;\left(U/L\right)}{Platelet\;count\;\left(10^{9}/L\right)\times \;\sqrt{ALT\;\left(U/L\right)}}$$

### 2.8. Statistical Data Analysis

Descriptive summary statistics are presented as group mean and standard deviation (SD) or median and interquartile range (IQR) for skewed data. Analyses of liver and kidney allograft SWS, φ as well as ALT level, proteinuria and eGFR were performed at four time points before and after treatment; linear mixed models with random intercept were used to account for repeated measures in subjects. Multiple imputation using chained equations and 30 imputed datasets (imputation method: predictive mean matching, package “mice”) [42] was used for the estimation of missing values for 15 individuals. For the imputation model, we used all outcome variables and information on sex, age, and time point. Model-based mean differences or mean estimates for different time points relative to baseline and 95% confidence interval (CI) are reported. All model-based estimates were adjusted for age. Correlations for repeated measures using the R package “rmcorr” [43] were calculated for the SWS and φ of the liver, the SWS and φ of the kidney allograft, FIB‑4 and APRI with 39 degrees of freedom, and 55 measures for 15 individuals. Associations between MRE and laboratory values were assessed by linear mixed models using bivariate analysis and multiple models with SWS as the outcome, adjusted for age. For the analysis of the correlation between SWS and φ of the liver, and fibrosis score and portal/periportal activity, the Spearman rank correlation coefficient was used. To evaluate the association between SWS and φ of the liver and lobular activity, which for our study only comprised two different values (0 or 1), we used the Mann–Whitney test using the formula: $r=\;\frac{Z}{\sqrt{n}}$, where *n* is the total sample size and *Z* is the Z-statistic of the Mann–Whitney test. Statistical analysis was performed with SPSS Statistics for Windows, version 25 (IBM, Armonk, NY, USA), GraphPad Prism v.6 (GraphPad software, La Jolla California USA), and R v4.0.2 (R Core Team, Vienna, Austria). A two-sided significance level of α = 0.05 was used. No adjustment for multiple testing was applied in this exploratory analysis; therefore, all *p*-values are descriptive only.

## 3. Results

### 3.1. Study Population

Of 1365 KTRs, 32 were identified during standard clinical follow-up as having chronic HCV infection. Fifteen of these complied with the inclusion and exclusion criteria (mean age (SD): 48 (13) years; seven female). Further descriptions are given in Table 1 and the study flow diagram in Figure 1.

### 3.2. Viral Response and Laboratory Values

**Figure 3:**
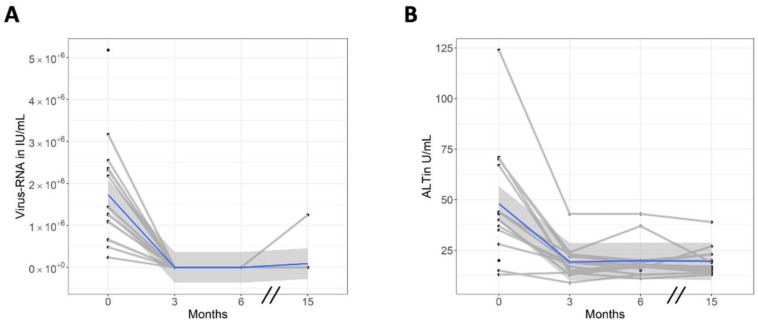
Laboratory results after antiviral treatment. (**A**) Viral load; complete viral clearance was achieved in all patients (*n* = 15) by 1 month after treatment initiation. The high value at 15 months (FU2) represents the single patient with viral relapse (first and second relapse 21 days after end of treatment (EOT) and 18 days after prolonged treatment are not shown); this patient had been HCV-negative for 3 months (EOT) and 6 months (FU1) after prolonged antiviral therapy (see text). (**B**) Alanine aminotransferase (ALT) was found to have decreased at the 3‑month measurement, corresponding to EOT, and it showed constant low values during follow-up; this included the patient with viral relapse.

The mean (SD) level of HCV RNA was 1.73 × 10^6^ (1.28 × 10^6^) IU/mL at baseline. The median interval between the initiation of therapy and viral clearance was 20 (IQR 11–28) days. Fourteen of 15 patients attained SVR12. In all patients who responded to DAA treatment, viral RNA was undetectable at each MRE follow-up (EOT, FU1 and FU2; Figure 3A). In one patient, a viral relapse occurred 21 days after EOT. Therefore, according to [27], the DAA treatment was extended to 24 weeks, leading to an undetectable viral load at FU1. In this patient, a second viral relapse was detected 18 days after the end of the second therapy period. Viral load was below the detection limit at EOT and FU1; however, the amount of viral RNA was high at FU2 (1.25 × 10^6^ IU/mL) owing to the second relapse (high-value dot in Figure 3A).

In comparison with baseline, ALT values decreased to normal levels directly after treatment at EOT (−29 U/L, CI = −41–−17; *p* < 0.001) and then remained stable until FU1 (−28 U/L, CI = −40–−16; *p* < 0.001) and FU2 (−29 U/L, CI = −40–−16; *p* < 0.001; see Figure 3B). Bilirubin values were not elevated at any time (all *p* >0.35). eGFR and proteinuria levels showed no substantial changes over the entire study period (*p* > 0.89 and *p* > 0.61). Details of laboratory values are provided in Table 2.

### 3.3. Tomoelastography—Liver

**Figure 4:**
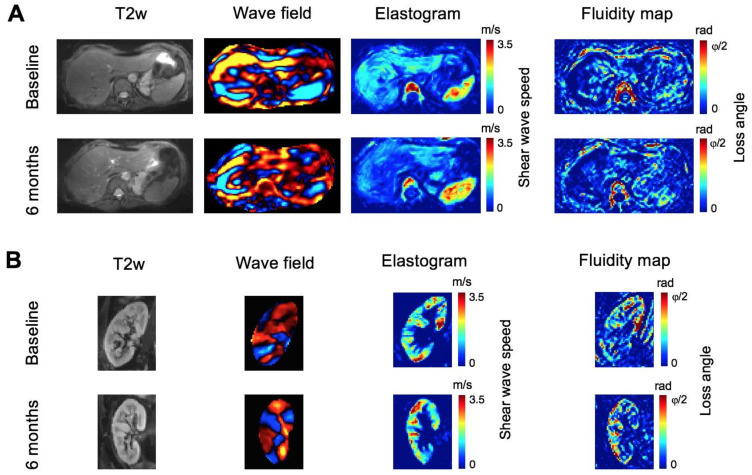
Representative MRE images. T2-weighted (T2w) images, tomoelastography wave-field images (50 Hz), elastograms and fluidity maps of the liver (**A**) and kidney allograft (**B**) at baseline and after 6 months (follow-up 1). Shear-wave speed and loss angle of the complex shear modulus increase in the liver after treatment with direct-acting antivirals, while both parameters increase in the kidney allograft.

**Figure 5:**
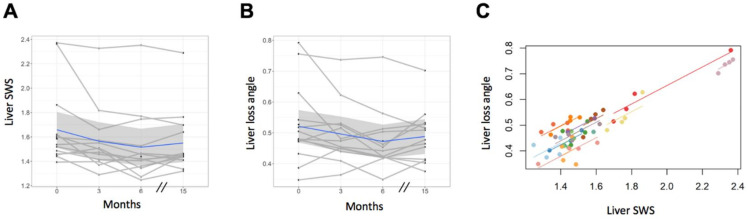
Results of liver tomoelastography after antiviral treatment. (**A**) Decrease in liver shear wave speed (SWS), accompanied by (**B**) reduced loss angle of the complex shear modulus (φ) at FU1, and SWS at FU2 compared with baseline (at FU1 for SWS *p* = 0.005, and for φ *p* = 0.038; at FU2 for SWS *p* = 0.47, and for φ *p* = 0.195). In one patient who suffered viral relapse with constant high values in (**A**,**B**), no substantial changes in SWS and φ were observed. (**C**) Analysis of repeated-measures correlation showed a strong correlation between SWS and φ (*r* = 0.81, CI = 0.67–0.90). EOT, end of treatment; FU1, follow-up 1; FU2, follow-up 2.

A decrease in liver stiffness and fluidity 3 months after EOT (FU1) was observed, as shown in Figure 4A. Compared with baseline, no changes were observed at EOT for liver SWS or liver φ (−0.10 m/s, CI = −0.21–0.01; *p* = 0.088/−0.03 rad, CI = −0.08–0.02; Figure 5A, B). Analysis of longitudinal measurements for the entire study cohort showed a decrease in liver SWS at FU1 (mean difference compared with baseline = −0.14 m/s, CI = −0.25–−0.04, *p* = 0.005) and at FU2 (mean difference = −0.11 m/s, CI = −0.23–−0.001, *p* = 0.047; Figure 5A). Accordingly, φ decreased at FU1 (−0.05 rad, CI = −0.10–−0.002, *p* = 0.038), while it remained unchanged at FU2 (−0.04, CI = −0.09–−0.01, *p* = 0.195; Figure 5B) compared with baseline. Both liver SWS and φ of the single patient who experienced viral relapse were persistently high at all four time points (high values in Figure 5A,B). Detailed results are given in Table 3.

Analysis of repeated measures correlation demonstrated a positive correlation between SWS and φ (*r* = 0.81, CI = 0.67–0.90; Figure 5C). No venous or bile-duct congestion in any patient was shown in the T2-weighted images.

### 3.4. Tomoelastography—Kidney Allograft

**Figure 6:**
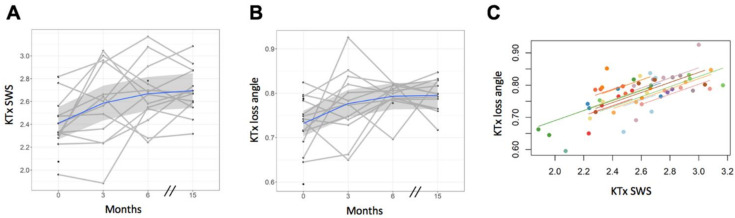
Results of kidney allograft tomoelastography after antiviral treatment. (**A**) Increase in kidney transplant shear wave speed (SWS) and (**B**) loss angle of the complex shear modulus (φ) at FU1 (SWS, *p* = 0.01; φ, *p* = 0.02) and FU2 (SWS, *p* = 0.004; φ, *p* = 0.02) compared with baseline. (**C**) Analysis of repeated-measures correlation shows a strong correlation between SWS and φ (*r* = 0.66, 95% CI = 0.44–0.81). EOT, end of treatment; FU1, follow-up 1; FU2, follow-up 2.

An increase in kidney allograft stiffness and fluidity 3 months after EOT (FU1) was observed, as shown in Figure 4B. No changes in SWS and φ were observed at EOT compared with baseline (mean differences from baseline for SWS = +0.19 m/s, CI = −0.04–0.41, *p* = 0.13; for φ = +0.04 rad, CI = −0.01–0.10, *p* = 0.17; Figure 6A,B). Compared with baseline, SWS and φ were increased in kidney allografts at FU1 (mean differences for SWS = +0.26 m/s, CI = 0.05–0.48, *p* = 0.01; for φ = +0.06 rad, CI = 0.007–0.11, *p* = 0.02) and at FU2 (mean differences for SWS = +0.30 m/s, CI = 0.08–0.52, *p* = 0.004; for φ = +0.06 rad, CI = 0.009–0.12, *p* = 0.02). These results are illustrated in Figure 6A,B, and details are presented in Table 3. Renal SWS and φ were positively correlated (*r* = 0.66, CI = 0.44–0.81; Figure 6C).

Renal SWS was not correlated with eGFR (*r* = −0.06, CI = −0.37–0.26) or with proteinuria (*r* = 0.03, CI = −0.29–0.34).

### 3.5. Histopathology

The following scores and grades were recorded: fibrosis (F0, *n* = 1; F1, *n* = 3; F2, *n* = 7 and F3, *n* = 2), inflammation in terms of portal and periportal activity (G0, *n* = 4; G1, *n* = 3; G2, *n* = 3 and G3, *n* = 3), and lobular activity (G0, *n* = 7; G1, *n* = 6; G2, *n* = 0 and G3, *n* = 0). Liver SWS and φ were positively correlated with fibrosis score (Spearman rank coefficient, *r* = 0.358 and 0.521). The inflammation scores were either not correlated at all or only weakly correlated with liver SWS and φ. Further details are provided in Table 4.

### 3.6. Serological Fibrosis Score

Compared with baseline, the APRI score showed a decrease at EOT (estimated means ± SD at baseline = 0.46 ± 0.19; at EOT = 0.24 ± 0.07; *p* < 0.001) and remained stable throughout follow-up (at FU1 = 0.28 ± 0.08, *p* = 0.002; at FU2 = 0.30 ± 0.21, *p* = 0.012). A similar observation was made for FIB‑4 score, where a decrease was found at EOT when compared with baseline (at baseline = 1.43 ± 0.66; at EOT = 1.19 ± 0.47; *p* = 0.59), but there was no difference in further follow-up (at FU1 = 1.33 ± 0.59; *p* = 0.945, at FU2 = 1.46 ± 1.08; *p* = 1.0). Liver SWS was moderately correlated with APRI score (*r* = 0.44, CI = 0.14–0.66) and was not correlated with FIB‑4 score (*r* = 0.12, CI = −0.20–0.42).

## 4. Discussion

In this prospective study, tomoelastography-based magnetic resonance elastography (MRE) was used for the first time to assess the short- and long-term outcome of DAA treatment in HCV-infected KTRs. In the following section, we focus upon our main findings in the liver and kidney.

### 4.1. Viscoelastic DAA Response in the Liver

We observed hepatic stiffness decrease after DAA treatment, in agreement with previous studies that used single-frequency MRE at 60 Hz [44] and ultrasound elastography (USE) [19,45,46,47,48,49,50]. These results suggest that reduction of the inflammation caused by DAA treatment is most probably the cause of the reduction in hepatic stiffness. Viral load and ALT, which is an indirect marker for HCV inflammatory activity [51], were markedly reduced in our cohort. In the literature, various mechanisms are discussed that might potentially link inflammation to liver stiffness [52,53,54]; among these, reversal of interstitial edema by DAA treatment might consistently explain the observed reduction of stiffness and fluidity at FU1 [52,53]. Another important element of liver stiffness is fibrosis. The antifibrotic effect of daclatasvir and sofosbuvir by downregulation of the tumor necrosis factor alpha (TNF‑α)/nuclear factor kappa B (NF‑κB) pathway [55] might have caused regression of liver fibrosis and contributed to the observed decrease in liver stiffness.

Interestingly, liver stiffness remained unchanged after DAA treatment in one patient only. Since this was the only patient who had a viral relapse, one might speculate about the ability of tomoelastography to predict, on the basis of the persistence of abnormally high liver stiffness, the success of DAA treatment at early time points. Notably, the non-responsiveness of this patient was not seen by HCV RNA load, ALT or serological fibrosis scores. The success of viral eradication in other studies has been reported, similarly to the present study, as being about 93% [10,13,56]. Thus, there is a need to identify those patients who will not respond to DAA therapy early, so that their therapy can be changed. Other studies using USE did not lead to a prediction of viral relapse based on liver stiffness [44,49,57,58]. However, USE has a limited field of view and cannot cover deep-lying tissues. By contrast, tomoelastography depicts the entire liver in 3D, which might explain the unique sensitivity of this method to DAA therapy.

Our observations on liver stiffness were paralleled by liver fluidity, which was introduced for the first time within the context of hepatic treatment response. The observed decrease in hepatic fluidity indicates a transition of the liver towards more solid-like behavior as a result of DAA treatment. We suspect that the decrease in fluidity was associated with changes in fluid–tissue interactions as a result of subsided inflammation after DAA treatment. Similarly, a positive correlation between hepatic inflammation and the damping ratio of the shear modulus has been reported in animal models [59]. A possible mechanism that links fluidity and inflammation is related to fluid pressure: sinusoidal hydrostatic pressure and lymphatic fluid are abnormally elevated in chronical hepatitis [60], thereby leading to a higher fluid content in the tissue and increased MRE fluidity. This could be an explanation of the observation in our study that histological fibrosis scores are better correlated with fluidity than with stiffness.

### 4.2. Viscoelastic DAA Response in Kidney Allografts

Proteinuria and serum creatinine, two markers for glomerular destruction [18], remained unchanged over the entire examination period after DAA treatment in our study. We obtained stable eGFR values over the course of 12 months, similarly to other studies [20,61,62,63]. Interestingly, we observed renal stiffening after DAA treatment, possibly as a result of altered kidney allograft perfusion. The high sensitivity of tomoelastography to functional and structural changes in renal tissue was demonstrated earlier in dysfunctional native [24,25] and transplanted [26] kidneys by the same technique used in the present study. In our earlier study [25], changes in renal stiffness outperformed eGFR in detecting patients with lupus nephritis at CKD stage 1—an early stage where renal function is not compromised. The observed stiffening of transplanted kidneys after DAA is in contrast to the previously reported stiffness decrease when renal function was compromised [24,25,63]. This is an encouraging result, as it suggests that DAA treatment does affect kidney allograft function, in agreement with other studies of DAA safety [18,27].

Fluidity, as a second mechanical parameter provided by tomoelastography, was used for the first time in this study for renal tissue characterization. We attribute the observed increase in fluidity to the possible elevation of renal perfusion after DAA treatment. The underlying mechanism might be similar to the observed effect in the liver, but with an opposite sign: here, regression of the disease results in an increased fluid content due restored renal perfusion. Although transient elastography has gained wide acceptance for determining liver stiffness [31], its use for renal transplants is not recommended. The lack of a visual control to visualize detailed anatomical structures of the kidney and the fixed measurement at a depth of 4 cm are the main limitations. Thus, only ultrasound elastography methods based on acoustic radiation force impulse (ARFI) imaging are currently included in the European Federation for Ultrasound in Medicine and Biology (EFSUMB) guidelines and only as a complementary tool for the diagnosis of chronic allograft nephropathy [64].

This study has several limitations. Owing to the limited numbers of KTRs with HCV infection, our sample size was small. There was no placebo control. Furthermore, liver biopsy was only performed at baseline because of its invasive nature. However, surrogate scores based on serological markers could be obtained at all times, and these showed a positive correlation between hepatic SWS and fibrosis. Another limitation was the inability to identify histopathological changes in kidney allografts. However, this study was intended to monitor the in vivo mechanical response of both the liver and kidney to DAA treatment without reference to ex vivo tissue examination.

In conclusion, tomoelastography is a non-invasive and quantitative multifrequency MRE technique to assess the response to DAA treatment and longitudinal changes in the liver and in kidney allografts without the need for contrast medium. Hepatic stiffness and fluidity decreased while kidney allograft stiffness and fluidity increased after virus elimination by DAA treatment, suggesting that this treatment restores mechanically sensitive structures and functions in the liver and kidney.

## Figures and Tables

**Table 1:** Demographics.

Characteristics	Kidney Allograft Recipients
Number of participants	15
Number of men	8
Number of women	7
Age in years	
Mean (SD)	48 (13)
Body mass index in kg/m^2^	
Mean (SD)	23.3 (4.5)
Time since kidney transplantation in years	
Mean (SD)	13.1 (6.9)

SD = standard deviation.

**Table 2:** Laboratory results.

Variable	Baseline	End of Treatment	FU1	FU2
	(*n* = 15)	(*n* = 13)	(*n* = 14)	(*n* = 13)
Viral parameters				
HCV-RNA (10^6^ × IU/mL)				
Mean (SD)	1.73 (1.28)	0 (0)	0 (0)	0.09 (0.33)
Mean difference vs baseline (95% CI)		−1.72 (−2.35–−1.08)	−1.72 (−2.36–−1.08)	−1.64 (−2.28–−1.01)
*p*-value, comparison with baseline		<0.001	<0.001	<0.001
Liver parameters				
ALT (U/L)				
Mean (SD)	48 (29)	19 (8)	20 (9)	20 (7)
*p*-value, comparison with baseline		<0.001	<0.001	<0.001
Billirubin (mg/dL)				
Mean (SD)	0.52 (0.21)	0.42 (0.16)	0.46 (0.24)	0.47 (0.35)
*p*-value, comparison with baseline		0.348	0.805	0.751
Renal parameters				
eGFR (mL/min/1.73m^2^)				
Mean (SD)	56 (17)	57 (19)	56 (18)	53 (20)
*p*-value, comparison with baseline		0.996	1.0	0.886
Proteinuria (mg/L)				
Mean (SD)	238 (275)	215 (287)	239 (235)	364 (500)
*p*-value, comparison with baseline		0.887	0.614	0.642

Mean difference vs. baseline. 95% CI is adjusted for age and based on multiple linear mixed models after multiple imputation of missing values. HCV RNA = hepatitis C virus ribonucleic acid; SD = standard deviation; CI = confidence interval; eGFR = estimated glomerular filtration rate; FU1 = follow-up 1 (3 months after end of treatment); FU2 = follow-up 2 (12 months after end of treatment).

**Table 3:** Multifrequency MRE.

Variable	Baseline	End of Treatment	FU1	FU2
	(*n* = 15)	(*n* = 13)	(*n* = 14)	(*n* = 13)
Liver				
Shear wave speed (ms)				
Mean (SD)	1.66 (0.31)	1.56 (0.27)	1.52 (0.28)	1.55 (0.26)
*p*-value, comparison with baseline		0.088	0.005	0.047
Phase angle of the loss modulus (rad)				
Mean (SD)	0.52 (0.12)	0.50 (0.10)	0.47 (0.09)	0.49 (0.09)
*p*-value, comparison with baseline		0.382	0.038	0.195
Kidney allograft				
Shear wave speed (ms)				
Mean (SD)	2.41 (0.25)	2.59 (0.35)	2.67 (0.27)	2.70 (0.21)
*p*-value, comparison with baseline		0.136	0.012	0.004
Phase angle of the loss modulus (rad)				
Mean (SD)	0.73 (0.06)	0.78 (0.08)	0.79 (0.03)	0.80 (0.04)
*p*-value, comparison with baseline		0.180	0.021	0.018

SD = standard deviation; CI = confidence interval; FU1 = follow-up 1 (3 months after end of treatment); FU2 = follow-up 2 (12 months after end of treatment).

**Table 4:** Correlation analysis between liver tomoelastography and histopathological scores.

Variable	Fibrosis Score	Portal/Periportal Activity	Lobular Activity
	(*n* = 13)	(*n* = 13)	*(n* = 13)
Shear wave speed	0.358 ^a^	−0.227 ^a^	0.040 ^b^
Phase angle of the loss modulus	0.521 ^a^	0.147 ^a^	0.277 ^b^

^a^ Spearman rank correlation coefficient. ^b^ Correlation coefficient based on the Mann–Whitney test.
